# Hepatitis E in liver biopsies from patients with acute hepatitis of clinically unexplained origin

**DOI:** 10.3389/fphys.2013.00351

**Published:** 2013-12-13

**Authors:** Uta Drebber, Margarete Odenthal, Stephan W. Aberle, Nadine Winkel, Inga Wedemeyer, Jutta Hemberger, Heidemarie Holzmann, Hans-Peter Dienes

**Affiliations:** ^1^Institute for Pathology, University of CologneKoeln, Germany; ^2^Department of Virology, Medical University of ViennaVienna, Austria; ^3^Department of Gastroneterology, University of CologneKoeln, Germany

**Keywords:** acute hepatitis, HEV, HEV genotype 3, immune response, FFPE material

## Abstract

Hepatitis E virus (HEV) is a small RNA virus and the infectious agent of hepatitis E that occurs worldwide either as epidemics in Asia caused by genotype 1 and 2 or as sporadic disease in industrialized countries induced by genotype 3 and 4. The frequency might be underestimated in central Europe as a cause of acute hepatitis. Therefore, we analyzed on liver biopsies, if cases of acute hepatitis with clinically unknown or obscure diagnosis were actually caused by the infection with HEV. We included 221 liver biopsies retrieved from the files of the institute of pathology during the years 2000 till 2010 that were taken from patients with acute hepatitis of obscure or doubtful diagnosis. From all biopsies RNA was extracted, prepared, and subjected to RT-PCR with specific primers. Amplified RNA was detected in 7 patients, sequenced and the genotype 3 could be determined in four of the seven of positive specimens from 221 samples. Histopathology of the biopsies revealed a classic acute hepatitis with cholestatic features and in some cases confluent necrosis in zone 3. Histology in a cohort of matched patients was less severe and showed more eosinophils. The analysis of the immune response by subtyping of liver infiltrating lymphocytes showed circumstantial evidence of adaptive immune reaction with CD 8 positive CTLs being the dominant lymphocyte population. In conclusion, in doubtful cases of acute hepatitis of unknown origin, HEV infection should be considered as etiology in central Europe. We demonstrate for the first time that the diagnosis can be made in paraffin-embedded liver biopsies reliably when no serum is available and also the genotype can be determined. The analysis of the immune response by subtyping of liver infiltrating lymphocytes indicates an adaptive mechanism suggesting in analogy with HAV, HBV and HCV that the virus itself is not cytopathic but liver damage is due to immune reaction.

## Introduction

Hepatitis E is an endemic disease in many developing countries and occurs both as sporadic cases but also in endemic outbreaks (Purcell and Emerson, 2010). According to a WHO report from 2012 there are over 20 million infections of hepatitis E every year with more than 60% taking place in east and south Asia in waterborne epidemic manifestations (Who Fact Sheet, 2012). In central Europe hepatitis E was regarded as a typical travel associated disease. In recent years, however, there are increasing numbers of reports of sporadic cases in industrialized countries UK (Dalton et al., 2008), the Netherlands (Herremans et al., 2007), France (Kamar et al., 2012), and Germany (Wichmann et al., 2008) of patients who acquired the disease at home. Almost all of the European patients were infected by genotype 3 whereas in cases from developing countries genotypes 1 and 2 could be isolated. In central Europe, animals as pigs, boars, and deer especially in rural areas could be identified as a reservoir of the infectious agent (Herremans et al., 2007; Wichmann et al., 2008). Reports from the Robert-Koch Institute, center of infectious diseases in Germany, showed an increased number of cases from the year 2001 (first year of registration of hepatitis E as an notifiable disease) up to 2012. The number increased from 30–338 patients with presumably a majority of autochthonous cases. The present data confirm that hepatitis E is a disease in Germany with a prevalence of antibodies of up to 14% in general population (Faber et al., 2012; Juhl et al., 2013).

In practice, clinicians are not always aware of this disease and many patients are biopsied with acute hepatitis of unknown clinical etiology. The diagnosis can be made by serology or more reliable by direct determination of HEV-RNA by RT- PCR. In order to see if the diagnosis can also be established in the liver biopsy and above that how many cases of acute hepatitis of clinical unknown etiology are caused by HEV, we examined liver biopsies from our file from 2000–2010. We assessed histopathology by the Ishak scoring system and isolated HEV-RNA from the paraffin sections and examined the virus genotype.

Additionally, we performed subtyping of infiltrating inflammatory cells because immunopathology of acute hepatitis E has so far not been documented in the tissue. From 221 patients with acute hepatitis we found seven cases which were HEV positive.

## Material and methods

### Patients, histology, and immunological characterization

221 cases with clinical diagnosis of hepatitis of unknown etiology were taken from the files of the Institute of Pathology, University Clinic of Cologne, Germany. All patients were negative for serology of HAV, HBV, HDV, HCV as well as EBV and CMV. Autoantibodies were not detected. Intake of alcohol and drugs were excluded clinically. Transaminase levels varied between 600 units per ml and 1400 units per ml. Bilirubin was above 5 mg/dl, gamma GT and alkaline phosphatase were below three times of upper normal limit. We matched this cohort of positive patients with the cohort of seven HEV- negative patients regarding age, gender and biochemical data. The liver biopsies were prepared according to standard protocols and stained for H and E, Prussian blue, Van Gieson for connective tissue as well as PASD and Gomori. Additionally, immunohistochemistry was performed for lymphocyte subtyping by applying antibodies against CDla, CD3, CD4, CD8, CD20, CD56, CD 57, CD68, and TIA according to the instructions of the manufacturers. The absolute number of positive infiltrating cells was determined by counting the cells in 20 areas with high power field (hpf) in the microscope including at least 10 portal tracts.

### RNA extraction from formalin-fixed, paraffin-embedded biopsies

Three 5–7 μm sections from formalin-fixed and paraffin-embedded samples were prepared and deparaffinized in xylene by incubation at 65°C for a total of 20 min, substituting xylene twice. After two washes with 100% ethanol, samples were lysed in 200 ml proteinase K buffer [500 mg/ml proteinase K (*Invitrogen*) 50 mM Tris-HCl pH 7.4 and 5mM EDTA pH 8] overnight. Subsequently, total RNA was extracted by phenol/chloroform and precipitated with 200 mM sodium acetate and isopropanol as previously described (Dries et al., 1999). RNA yield was quantified by A260/280 measurement using a ND-1000 NanoDrop spectrophometer (NanoDrop, Wilmington, DE).

### HEV RNA determination by real time PCR

Extracts of total RNA (10–50 ng) were reverse transcribed in a 10 μl volume using Superscript reverse transcriptase (*Invitrogen*, Darmstadt, Germany), 5 μmol random primer (Roche Diagnostics, Mannheim, Germany), 1x First strand buffer (*Invitrogen*), 0.5 micro mol dNTP, and 0.5 U RNase Inhibitor (Roche Diagnostics). Incubation was performed according to the recommendations of *Invitrogen*. Then 1 μl of the reverse transcription reaction was used in each of the real-time PCR assays by means with the TaqMan® RNA Assay-Kit (Applied Biosystems) following the manufacturer's instructions. The sequence of primers and a HEV specific LNA probe were modified according to Jothikumar (Jothikumar et al., 2006) and are listed in Table 1.

**Table 1:** **Primers and Probes**.

**Oligonucleotide**	**Sequence**	**Assay**
Hep-E-F	CGGTGGTTTCTGGGGTGAC	Real-time PCR (primer)
Hep-E-R	GGRTTGGTTGGATGAATATAGG	Real-time PCR (primer)
Hep-E-P	Fam-TGATTCTCAGCCCTTCGC-BHQ–1	Real-time PCR (probe)
β-actin-F	TTGGCAATGAGCGGTTCCGCTG	Real-time PCR (primer)
β-actin-R	CACGTCACACTTCATGATGGAG	Real-time PCR (primer)
β-actin-P	Fam-tccagccttccttcctgggcatg-BHQ–1	Real-time PCR (probe)
HEV-M13uF	TGTAAAACGACGGCCAGTCTACGGTGGTTTCTGGGGTGAC	Sequencing
HEV-M13rR	CAGGAAACAGCTATGACCGGRTTGGTTGGATGAATATAGG	Sequencing

Real-time PCR amplification was carried out by a two-step incubation protocol (20 s of each:a 95°C step for denaturation and a 60°C step for annealing and synthesis). PCR assays of all samples were performed in triplicates on a CFX 96 thermocycler from Biorad Laboratories (Munich, Germany).

In order to identify the HEV genotype of specimens in which HEV-positive amplification was shown, a qualitative HEV-PCR was performed using M13 universe or reverse tailed HEV-specific primers, respectively, (Table 1) (10). The corresponding M13 primer set was then taken to sequence both directions of the amplicons using the BigDyeTerminator v 3.1 Cycle Sequencing Kit (Applied Biosystems). Sequence analysis was then performed by capillary electrophoresis using a ABI 3730 platform (Life Technologies, Darmstadt, Germany).

## Results

### Identification of HEV-RNA in the tissue

**Figure 1:**
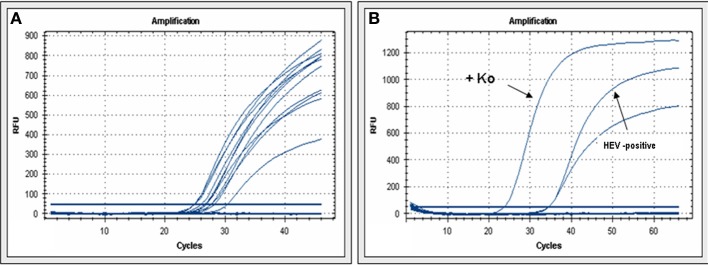
**HEV real-time PCR**. **(A)** β-actin real-time PCR demonstrates that extracted RNA is accessible for PCR. **(B)** Examples of real-Time PCR for HEV-cDNA from a positive reference (Ko) or from one of the human biopsies with acute hepatitis.

From a total of 221 biopsies representing histology of acute hepatitis of unknown clinical etiology RNA was analysed for the HEV genome by high sensitivity real-time PCR. RNA quality was proven by intron-spanning β-actin amplication (Figure 1A). By HEV specific real-time PCR seven liver biopsies were shown to be positive for HEV genomic RNA (Figure 1B). Subsequent sequence analysis could demonstrate that four patients had an infection with HEV genotype 3.

### Histopathologic features of acute HEV infection

**Figure 2:**
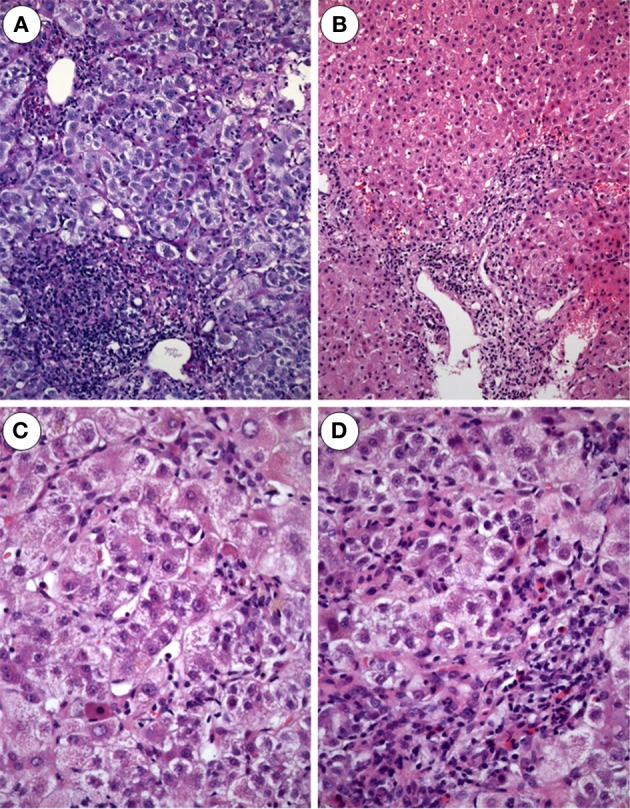
**Acute hepatitis E. (A)** Expanded portal tract with dense inflammatory infiltrates mostly lymphocytes. Bile ducts display mild accompanying cholangitis (H and E × 100). **(B)** Acute hepatitis E with enlarged portal tract densely infiltrated by lymphocytes and some PMN leukocytes as well as some spotty necroses in the lobule (H and E × 80). **(C)** Acute hepatitis E with areas of spotty necrosis, aptotic bodies and infiltrates of lymphocytes, Kupffer cells and few polymorphnuclear leukocytes (H and E m× 240). **(D)** Biopsy from a patient with acute hepatitis E: the lobule shows foci of spotty necrosis, ballooning of hepatocytes and infiltrates with lymphocytes and polymorphnuclear leukocytes (H and E × 240)

Next, the seven cases of HEV positive cases were matched with a group of biopsies representing acute hepatitis from patients of similar age, gender and biochemistry but negative HEV-RNA. The mean age of the patients was 58 years (40–78y) (Table 2). The histopathology of HEV positive biopsies showed the classical picture of acute hepatitis with spotty necrosis and portal expansion with inflammatory infiltrates mostly lymphocytes, but also a considerable number of polymorphnuclear leucocytes (Figure 2). Bile ducts were involved with cholangitis and lymphocytic infiltrates of biliary epithelia. Portal tracts were devoid of connective tissue and the features of chronic hepatitis were absent. In the lobules spotty necroses were present in all biopsies to a varying degree. Four of the biopsies showed confluent necrosis and in one patient central necrosis with portal bridging was observed. Interestingly, cholestasis was noted with bile plugs in dilated canaliculi but also with intracellular bile pigment. The staining for iron was negative in all cases.

**Table 2:** **Characteristics of Patients with Acute Hepatitis E**.

**No**.	**Age**	**Gender**	**Transaminases**	**Stays in abroad**	**EBV**	**CMV**
1	60	Female	AST 1300 U/L, ALT 2400 U/L	n.d.	Negative	Negative
2	61	Male	>1000 U/L	n.d.	Negative	Negative
3	65	Female	AST 2200 U/L, ALT 2300 U/L	n.d.	Negative	IgM: positive PCR: Negative
3	78	Female	max: AST 1573 U/L, ALT 1586 U/L at the date of bíopsy: AST 343 U/L, ALT 724 U/L	n.d.	Negative	IgM: positve/Negative PCR: Negative
4	40	Male	AST 541 U/L, ALT 474 U/L	Greece	Negative	Negative
5	42	Female	max: ALT > 600 U/L at the date of bíopsy: ALT 70 U/L	n.d.	Negative	Negative
6	57	Male	n.d.	n.d.	Negative	Negative

In the HEV-negative group, we found more eosinophilic leucocytes and the incidence of confluent necrosis was less frequent (see Table 3). Only one biopsy displayed bridging hepatic necrosis. Table 3 summarizes the comparison of HEV histology characteristics.

**Table 3:** **Histopathology in 7 HEV patients and matched cohort of 7 non-HEV patients scoring according to the hepatitis activity index (HAI)**.

	**HAI Score (Mean Values)**	
	**HEV group**	**Non-HEV group**
Confluent necrosis	13	10
Spotty necrosis, apoptosis, and local inflammation	25	25
Portal inflammation	18	8

Comment: Cholestasis and reactive cholangitis was more prominent in patients with HEV. In the non-HEV group 3 of 7 showed the presence of many eosinophils in portal tracts and lobules.

### Subtyping of the infiltrating inflammatory cells by immunohistochemistry

In order to see if the analysis of the infiltrating inflammatory infiltrates especially the lymphocytes could give some information of underlying immune mechanisms we performed immunohistological subtyping of the lymphocytes.

The exact data are given in Table 4 with the absolute number of positive cells when counted in 20 high power fields. The numbers give the mean range for each cell-population.

**Table 4:** **The number of liver infiltrating inflammatory cell^s#^**.

**Immune cell marker epitope**	**HEV Biopsies**	**Matched biopsies**
CDla	82 (*SD* = 9.6)	95 (*SD* = 7.5)
CD3	420 (*SD* = 14.7)	370 (*SD* = 15.5)
CD4	138 (*SD* = 11.9)	122 (*SD* = 9.1)
CD8	287 (*SD* = 20.3)	230 (*SD* = 12.1)
CD20	65 (*SD* = 6.5)	55 (*SD* = 6.7)
CD56	29 (*SD* = 3.3)^*^	49 (*SD* = 5.5)^*^
CD57	25 (*SD* = 3.8)^*^	52 (*SD* = 7.1)^*^
CD68	71 (*SD* = 9.7)	75 (*SD* = 7.5)
TIA	97 (*SD* = 9.2)	102 (*SD* = 12.6)

Given in absolute numbers per 20 hpf's at a mean range and standard deviation (SD) in brackets.

* Differences in number of marker cells observed in HEV positive vs. HEV negative tissue was significant (p < 0.05).

T-lymphocytes, positive for CD 3 made up the majority of the infiltrating cells. CD 8 positive lymphocytes were the second most numerous cellpopulation with higher numbers in HEV patients but not statistically significant from non-HEV patients. CD 4 positive lymphocytes made up the third frequent cell population whereas B-lymphocytes, positive for CD 20, were less numerous. There was quite a highportion of CD 68 positive macrophages.in both HEV-positive and negative acute hepatitis. However, in non-HEV patients we observed asignificant trend for a higher NK activity (Table 4).

## Discussion

Reports on autochthonous hepatitis E are increasing in industrialized countries (Purcell and Emerson, 2010; Who Fact Sheet, 2012). Transmission of genotype 3 has been implicated as the infectious agent also from animals like pigs, deer and boars which seem to be the reservoir and the consumption of meat of these animals has been identified as the source of infection. The prevalence of the infection in the general population has been estimated between one and 14 percent in several countries of Europe (Wichmann et al., 2008; Kamar et al., 2012; Juhl et al., 2013). In clinical practice, without a history of traveling to developing countries the hepatitis is often not diagnosed correctly thus the patients are biopsied to evaluate the cause of acute hepatitis of unexplained etiology.

In order to see how many biopsies from patients with clinically unknown etiology maybe due to infection with HEV and also to evaluate if the virus can be detected in the liver tissue, we performed an analysis on liver biopsies from the files of our institute between the years 2000–2010. We selected 221 biopsies with the clinical diagnosis of acute hepatitis of unknown origin. Infection with other hepatitis viruses as HAV, HBV, HDV, HCV, CMV, and EBV was excluded as well as consumption of drugs, alcohol and previous travels to endemic areas. In our cases the serology of anti HEV was not available. Seven of the 221 biopsies proved to contain HEV-RNA after extraction of the RNA and application of real-time-PCR with specific primers. Whereas Gupta et al. suggested that immunohistology might be superior to RNA based detection methods (Gupta et al., 2012), our PCR approach benefits from a primer design allowing detection of low copy HEV numbers and the differentiation of HEV gentoypes. Furthermore, low amplicon length (69 bp) reduces amplification problems of fragmented nucleic acids derived from FFPE material. Although Real Time PCR has the advantage to be comparable in sensivity to nested PCR (Ratcliff et al., 2007; Drebber et al., 2011), formalin artifacts due to fragmentation and polymerase synthesis errors are limitations of the pathogen detection in archived material. Multiple polymerase reading errors due to formalin caused base mismatching might have also been the reason for three HEV amplicons failing to be sequenced. However, the virus could be specified as genotype 3 in four of seven patients after sequencing. This is in accordance with other reports from UK (Dalton et al., 2008), France (Kamar et al., 2012), Netherlands (Herremans et al., 2007) and Germany (Wichmann et al., 2008).

The histopathology of acute hepatitis E has been reported in animals and few liver biopsies from patients with acute infection mostly in endemic areas. There is less information on histology in hepatitis E when compared to other virotypes of hepatitis. A short description is given in the textbook of MacSween by These, however, referring to infection with all genotypes of HEV (Theise et al., 2011). Only few reports deal with histopathology of the indigenous form of hepatitis E in Europe namely infection with genotype 3 (Malcolm et al., 2007; Peron et al., 2007). The study of Malcolm et al describes histopathology of sporadic hepatitis E in more details and underlines the cholestatic appearance of the lesion with ductular proliferation and cholangiolitis in the portal tracts (Malcolm et al., 2007). So cholangitic-cholestatic features seem to be characteristic for acute hepatitis E. Another case report published by Wendum et al. (2005) of acute sporadic hepatitis E even showed lymphocytic destructive cholangitis. Our cases confirm this observation with prominent cholestasis and cholangitis in four of the seven biopsies. We could not find any pathognomonic features, but a histopathology that resembles a classic hepatitis with spotty necrosis, extending to confluent necrosis in four of the seven biopsies. In the non-HEV group confluent necrosis was less severe, cholestasis was also less prominent and in three biopsies the presence of eosinophils was obvious. In clinical practice HEV infection can be reliably established by serology with IgM and IgG antibodies and PCR techniques in the serum. Our reports demonstrate for the first time that the diagnosis can be made also in the liver tissue when no serum is available. Extracted RNA can be sequenced to determine the genotype which turned out to be genotype 3 in all our cases confirming other reports from industrialized areas as the disease as an autochthonous infection. The immunopathology of liver damage seems to be T-cell mediated with CD 8 positive CTL's being the major population of infiltrating lymphocytes. The subtyping of inflammatory infiltrating cells by immunohistochemistry (see Table 3) showed CD 3/CD 8 positive lymphocytes as a predominant population suggesting an adaptive immune response to the virus as a major mechanism of defense and liver damage and supporting results obtained from analysis of peripheral blood lymphocytes (Suneetha et al., 2012). In the non-HEV group the number of CD 56/CD 57 lymphocytes was higher than in the patients perhaps due to another mechanism. In combination with the presence of eosinophils the findings suggest that these patients may have undergone drug induced liver injury without reporting drug intake which can be an important differential diagnosis to acute hepatitis E (Davern et al., 2011).

In conclusion we show that in cases of clinically unexplained acute hepatitis in central Europe the cause may be HEV. For the first time we document that the diagnosis can be made reliably on liver biopsies by PCR. Furthermore, this is the first report on histopathology of HEV infection exclusively- by genotype 3. Immunopathology with predominant CD 3/CD 8 positive lymphocytes indicate an adaptive immune response as defense and the cause of liver damage.

## Author contributions

Conceived and designed the experiments: Heidemarie Holzmann, Hans-Peter Dienes. Performed the experiments: Margarete Odenthal, Inga Wedemeyer, Stephan W. Aberle, Jutta Hemberger. Analyzed the data: Heidemarie Holzmann, Uta Drebber, Inga Wedemeyer, Nadine Winkel, Stephan W. Aberle. Contributed reagents/materials/analysis tools: Hans-Peter Dienes, Margarete Odenthal. Wrote the paper: Heidemarie Holzmann, Hans-Peter Dienes. Performed the illustrations of the data: Margarete Odenthal, Uta Drebber.

### Conflict of interest statement

The authors declare that the research was conducted in the absence of any commercial or financial relationships that could be construed as a potential conflict of interest.

## References

[B1] Dalton H. R. Stableforth W. Thurairajah P. Hazeldine S. Remnarace R. Usama W. (2008). Autochthonous hepatitis E in Southwest England: natural history, complications and seasonal variation, and hepatitis E virus IgG seroprevalence in blood donors, the elderly and patients with chronic liver disease. Eur. J. Gastroenterol. Hepatol. 20, 784–790 10.1097/MEG.0b013e3282f5195a 18617784

[B2] Davern T. J. Chalasani N. Fontana R. J. Hayashi P. H. Protiva P. Kleiner D. E. (2011). Acute hepatitis E infection accounts for some cases of suspected drug-induced liver injury. Gastroenterology 141, 1665-72.e1–1665-72.e9 10.1053/j.gastro.2011.07.051 21855518 PMC3654540

[B3] Drebber U. Hardt A. Dienes H. P. Odenthal M. (2011). Cytomegalovirus. Pathological-anatomical manifestations and detection methods. Pathologe 32, 418–427 10.1007/s00292-011-1449-8 21792604

[B4] Dries V. Von Both I. Muller M. Gerken G. Schirmacher P. Odenthal M. (1999). Detection of hepatitis C virus in paraffin-embedded liver biopsies of patients negative for viral RNA in serum. Hepatology 29, 223–229 10.1002/hep.510290118 9862870

[B5] Faber M. S. Wenzel J. J. Jilg W. Thamm M. Hohle M. Stark K. (2012). Hepatitis E virus seroprevalence among adults, Germany. Emerging Infect. Dis. 18, 1654–1657 10.3201/eid1810.111756 23018055 PMC3471611

[B6] Gupta P. Jagya N. Pabhu S. B. Durgapal H. Acharya S. K. Panda S. K. (2012). Immunohistochemistry for the diagnosis of hepatitis E virus infection. J. Viral Hepat. 19, e177–e183 10.1111/j.1365-2893.2011.01498.x 22239516

[B7] Herremans M. Vennema H. Bakker J. Van Der Veer B. Duizer E. Benne C. A. (2007). Swine-like hepatitis E viruses are a cause of unexplained hepatitis in the Netherlands. J. Viral Hepat. 14, 140–146 10.1111/j.1365-2893.2006.00786.x 17244254

[B8] Jothikumar N. Cromeans T. L. Robertson B. H. Meng X. J. Hill V. R. (2006). A broadly reactive one-step real-time RT-PCR assay for rapid and sensitive detection of hepatitis E virus. J. Virol. Methods 131, 65–71 10.1016/j.jviromet.2005.07.004 16125257

[B9] Juhl D. Baylis S. A. Blumel J. Gorg S. Hennig H. (2013). Seroprevalence and incidence of hepatitis E virus infection in German blood donors. Transfusion. [Epub ahead of print]. 10.1111/trf.12121 23441647

[B10] Kamar N. Bendall R. Legrand-Abravanel F. Xia N. S. Ijaz S. Izopet J. (2012). Hepatitis E. Lancet 379, 2477–2488 10.1016/S0140-6736(11)61849-7 22549046

[B11] Malcolm P. Dalton H. Hussaini H. S. Mathew J. (2007). The histology of acute autochthonous hepatitis E virus infection. Histopathology 51, 190–194 10.1111/j.1365-2559.2007.02756.x 17650215

[B12] Peron J. M. Danjoux M. Kamar N. Missoury R. Poirson H. Vinel J. P. (2007). Liver histology in patients with sporadic acute hepatitis E: a study of 11 patients from South-West France. Virchows Arch. 450, 405–410 10.1007/s00428-007-0382-y 17333266

[B13] Purcell R. H. Emerson S. U. (2010). Hidden danger: the raw facts about hepatitis E virus. J. Infect. Dis. 202, 819–821 10.1086/655900 20695795 PMC2941993

[B14] Ratcliff R. M. Chang G. Kok T. Sloots T. P. (2007). Molecular diagnosis of medical viruses. Curr. Issues Mol. Biol. 9, 87–102 17489437

[B15] Suneetha P. V. Pischke S. Schlaphoff V. Grabowski J. Fytili P. Gronert A. (2012). Hepatitis E virus (HEV)-specific T-cell responses are associated with control of HEV infection. Hepatology 55, 695–708 10.1002/hep.24738 22006345

[B16] Theise N. Bodenheimer H. Feerel L. (2011). Acute and chronic viral hepatitis, in MacSween's Pathology of the Liver, eds Burt A. Portman B. Ferrel L. (New York, NY: Elsevier, Churchill Livingston), 361–401

[B17] Wendum D. Nachury M. Yver M. Lemann M. Flejou J. F. Janin A. (2005). Acute hepatitis E: a cause of lymphocytic destructive cholangitis. Hum. Pathol. 36, 436–438 10.1016/j.humpath.2005.01.006 15892007

[B18] Who Fact Sheet H. E. (2012). Hepatitis E. WHO Fact Sheet No:280, Geneva

[B19] Wichmann O. Schimanski S. Koch J. Kohler M. Rothe C. Plentz A. (2008). Phylogenetic and case-control study on hepatitis E virus infection in Germany. J. Infect. Dis. 198, 1732–1741 10.1086/593211 18983248

